# 1,4-Disubstituted-1,2,3-Triazole Compounds Induce Ultrastructural Alterations in *Leishmania amazonensis* Promastigote: An in Vitro Antileishmanial and in Silico Pharmacokinetic Study

**DOI:** 10.3390/ijms21186839

**Published:** 2020-09-18

**Authors:** Fernando Almeida-Souza, Verônica Diniz da Silva, Gabriel Xavier Silva, Noemi Nosomi Taniwaki, Daiana de Jesus Hardoim, Camilla Djenne Buarque, Ana Lucia Abreu-Silva, Kátia da Silva Calabrese

**Affiliations:** 1 Pós-graduação em Ciência Animal, Universidade Estadual do Maranhão, São Luís 65055-310, Brazil; 2 Laboratório de Imunomodulação e Protozoologia, Instituto Oswaldo Cruz, Fiocruz, Rio de Janeiro 21040-900, Brazil; daianahardoim@gmail.com (D.d.J.H.); calabrese@ioc.fiocruz.br (K.d.S.C.); 3 Laboratório de Síntese Orgânica, Pontifícia Universidade Católica, Rio de Janeiro 22451-900, Brazil; veronk.d1niz@gmail.com (V.D.d.S.); camilla.buarque@gmail.com (C.D.B.); 4 Faculdade de Ciência e Tecnologia, Universidade Nova de Lisboa, 2825-149 Caparica, Portugal; 5 Rede Nordeste de Biotecnologia, Universidade Federal do Maranhão, São Luís 65080-805, Brazil; xaviersilva.g@gmail.com; 6 Núcleo de Microscopia Eletrônica, Instituto Adolfo Lutz, São Paulo 01246-000, Brazil; noemi.taniwaki@ial.sp.gov.br

**Keywords:** cytotoxicity, transmission electron microscopy, leishmaniasis, treatment, ADME, toxicity

## Abstract

The current standard treatment for leishmaniasis has remained the same for over 100 years, despite inducing several adverse effects and increasing cases of resistance. In this study we evaluated the in vitro antileishmanial activity of 1,4-disubstituted-1,2,3 triazole compounds and carried out in silico predictive study of their pharmacokinetic and toxicity properties. Ten compounds were analyzed, with compound **6** notably presenting IC_50_: 14.64 ± 4.392 µM against promastigotes, IC_50_: 17.78 ± 3.257 µM against intracellular amastigotes, CC_50_: 547.88 ± 3.256 µM against BALB/c peritoneal macrophages, and 30.81-fold selectivity for the parasite over the cells. It also resulted in a remarkable decrease in all the parameters of in vitro infection. Ultrastructural analysis revealed lipid corpuscles, a nucleus with discontinuity of the nuclear membrane, a change in nuclear chromatin, and kinetoplast swelling with breakdown of the mitochondrial cristae and electron-density loss induced by 1,4-disubstituted-1,2,3-triazole treatment. In addition, compound **6** enhanced 2.3-fold the nitrite levels in the *Leishmania*-stimulated macrophages. In silico pharmacokinetic prediction of compound **6** revealed that it is not recommended for topical formulation cutaneous leishmaniasis treatment, however the other properties exhibited results that were similar or even better than miltefosine, making it a good candidate for further in vivo studies against *Leishmania* parasites.

## 1. Introduction

Leishmaniasis is a complex of infectious diseases caused by protozoa of the *Leishmania* genus. It is transmitted through the bite of female sandflies, and its clinical manifestations include cutaneous, mucosal and visceral forms, with the third presenting considerable rates of morbidity and mortality [1,2]. Even today, it continues one of the most important global parasitic diseases, affecting millions of people, especially in developing countries [3]. The World Health Organization (WHO) estimates there are 12 million cases of visceral leishmaniasis worldwide, with 300,000 new cases and more than 20,000 deaths per year, while there have been 1 million new cases of cutaneous leishmaniasis reported in the last five years. More than 1 billion people live at risk of infection, and 90% of registered cases of leishmaniasis occur in developing countries [4].

The current treatment is still based on pentavalent antimonials, which have been in use since 1912, and like the other drugs used in leishmaniasis treatment, such as amphotericin B, pentamidine and miltefosine, induce several adverse drug effects [5,6,7,8]. In general, treatment is expensive and (with the exception of miltefosine, which is administrated orally) causes discomfort to patients due to parenteral administration for prolonged periods. These characteristics, associated with the increasing number of cases of resistance to current treatments, immunosuppressed patients (HIV coinfections and malnourished individuals, for example) and those with hepatic and renal disorders, showing the necessity for research into new therapy options that are more efficient and non-toxic [9,10].

Triazoles are important heterocycles of synthetic origin involved in several industrial applications, such as agrochemistry, material sciences, and the synthesis of new drugs [11,12]. 1,2,3-triazole is an important pharmacophoric group present in several heterocyclic compounds [13], and covers a wide range of biological applications, such as anti-tubercular [14], antimicrobial [15], anticonvulsant [16], antiviral [17], and anticancer [18] activities, among others. It can be obtained by several synthetic routes; however, the click chemistry reaction, also known as copper-catalyzed alkyne-azide cycloaddition, is a powerful tool in chemical medicine for the supply of highly versatile 1,2,3-triazole scaffolds due to its simplicity, robustness, and applicability [11,19,20].

The triazole compound family is also known for its potential activity against fungi [21] and trypanosomatids, such as parasites of the genus *Leishmania* [22,23]. We recently carried out the design, synthesis, and structural characterization of new 1,4-disubstituted-1,2,3-triazole compounds by copper-catalyzed azide-alkyne click chemistry reaction [24]; however, its antileishmanial potential has not yet been elucidated. Triazole compounds were planned to correlate the effect of the exchange of functional groups of aldehydes by classical privileged groups such as sulphonylhydrazones, hydrazones, and coumarin with anticancer and antileishmanial activity [24,25,26]. These groups can also act in synergism with the 1,2,3-triazole nucleus and potentiate the pharmacological activity. The triazole group has interesting physical-chemical and chemical properties that can mimic the characteristics of different functional groups [13,24].

In the present study, we evaluated the in vitro antileishmanial activity of the 1,4-disubstituted-1,2,3-triazole compounds, demonstrating the ultrastructural alterations the treatment induced in *Leishmania amazonensis* promastigotes. In addition, we performed an in silico predictive study of the drug-likeness, pharmacokinetics properties, and toxicity of the triazole compounds that exhibited the best antileishmanial activity.

## 2. Results

### 2.1. Activity of 1,4-Disubstituted-1,2,3-Triazole against L. amazonensis Promastigote and Intracellular Amastigote Forms

**Figure 1:**
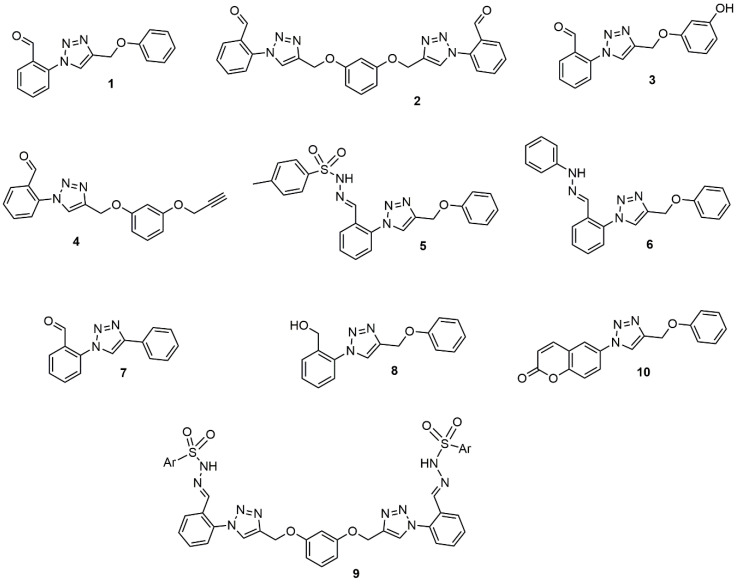
Chemical structure of the 1,4-disubstituted-1,2,3-triazoles derivatives analyzed.

The IC_50_ results of the activity against promastigote and intracellular amastigote forms of the 1,4-disubstituted-1,2,3-triazole compounds (Figure 1) are shown in Table 1. The trial assay against *L. amazonensis* promastigote revealed that compounds **2** and **5** demonstrated greater activity, with 50% inhibitory concentration (IC_50_) values below 10 μM. Compounds **4** and **6** presented intermediate activity, with IC_50_ around 15 μM. The other compounds, **1**, **3**, and **7**, presented activity above 50 μM or did not exhibit any such activity even at the maximum analyzed concentration, such as compounds **8**, **9**, and **10**.

**Figure 2:**
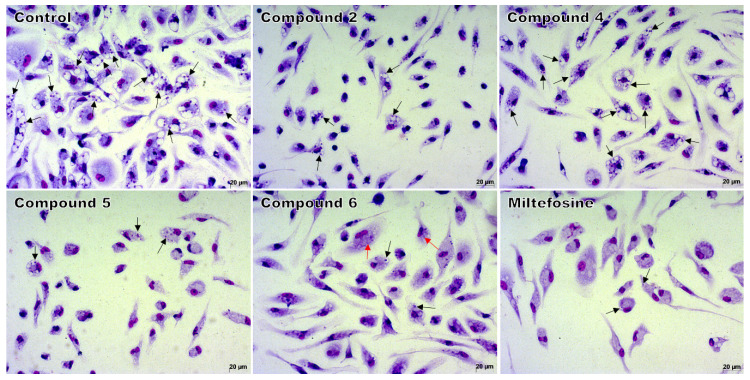
Light microscopy of macrophages infected and treated with 1,4-disubstituted-1,2,3-triazole compounds **2**, **4**, or **5** at 37.5 μM; with compound **6** at 18.7 μM; or with miltefosine at 25 μM. Intracellular amastigotes (black arrows) and remains of amastigotes (red arrows) inside macrophages. Giemsa, 40× objective. The images are representative of two independent experiments performed in quadruplicate.

The compounds that exhibited greater (**2** and **5**) and intermediate activity (**4** and **6**) against promastigote forms were selected for evaluation against the intracellular amastigote. Compound **6** exhibited the lowest IC_50_, maintaining an active concentration similar to that obtained against the promastigote form. Compounds **2** and **5**, which had the best result against promastigotes, showed a more than four-fold increase in IC_50_ value against intracellular amastigote forms when compared to their IC_50_ values against promastigotes. Compound **4** had the worst activity among the compounds evaluated against intracellular amastigote, with a 9.5-fold increase in IC_50_ value compared to the promastigote forms. The decrease in intracellular amastigotes can be visually observed in the light microscopy images (Figure 2).

**Figure 3:**
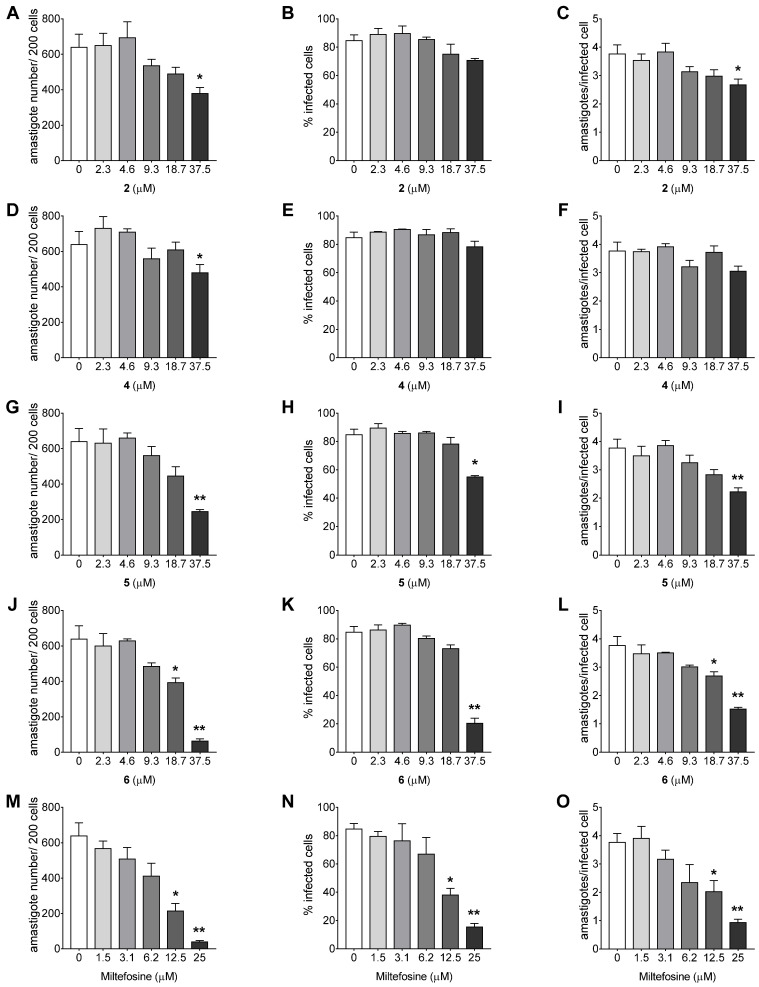
Activity of 1,4-disubstituted-1,2,3-triazole compounds against *Leishmania amazonensis* intracellular amastigotes. Parameters of infection of BALB/c peritoneal macrophages infected with *L. amazonensis* and treated with triazole compounds (**A**–**L**) or mltefosine (**M**–**O**) for 24 h. The data represent mean ± standard deviation of two independent experiments performed in quadruplicate. * *p* < 0.05 and ** p < 0.01 when compared with the control group by Kruskal–Wallis followed by Dunn’s multiple comparisons test.

The comparison of the parameters of infection of the treated infected cells with untreated infected cells (Figure 3A–O) corroborates the activity against intracellular amastigote results. Compound **4** statistically decreased the number of amastigotes per 200 cells (*p* = 0.0481, Figure 3D) at 37.5 μM only, and did not significantly alter the other parameters of infection at any of the concentrations evaluated. Compound **2** decreased the number of amastigotes per 200 cells (*p* = 0.0193, Figure 3A) and amastigotes per infected cell (*p* = 0.0102, Figure 3C) at 37.5 μM, whereas compound **5** reduced all the three parameters of infection at 37.5 μM, especially the number of amastigotes per 200 cells (*p* = 0.0065, Figure 3G) and amastigotes per infected cell (*p* = 0.0070, Figure 3I). Compound **6** altered all the three parameters of infection at 37.5 μM with a remarkable reduction in the number of amastigotes per 200 cells (*p* = 0.0028, Figure 3J) and was still able to reduce the number of amastigotes per 200 cells (*p* = 0.0375, Figure 3J) and amastigotes per infected cell (*p* = 0.0333, Figure 3L) at 18.7 μM, demonstrating the best activity of the compounds analyzed. Miltefosine exhibited reduction of all three parameters of infection at 25 and 12.5 μM (*p* = 0.0024 and *p* = 0.0123, Figure 3M; *p* = 0.0013 and *p* = 0.0313, Figure 3N; *p* = 0.0036 and *p* = 0.0321, Figure 3O).

### 2.2. Cytotoxicity and Selectivity Index (SI) of 1,4-Disubstituted-1,2,3-Triazole Compounds

Compound **5** presented the highest cytotoxicity among the tested compounds, with all the other compounds exhibiting CC_50_ values above 100 μM. Compound **4** showed the lowest SI value among the analyzed compounds due to low activity against the intracellular amastigotes, and was more toxic to the cell than to the intracellular amastigote. Compound **6** exhibited the lowest cytotoxicity among the analyzed compounds and, as well as having the best activity against intracellular amastigotes, resulted in the highest SI—higher even than miltefosine (Table 1).

### 2.3. Ultrastructural Alterations in L. amazonensis Promastigotes Treated with 1,4-Disubstituted-1,2,3-Triazole Compounds

**Figure 4:**
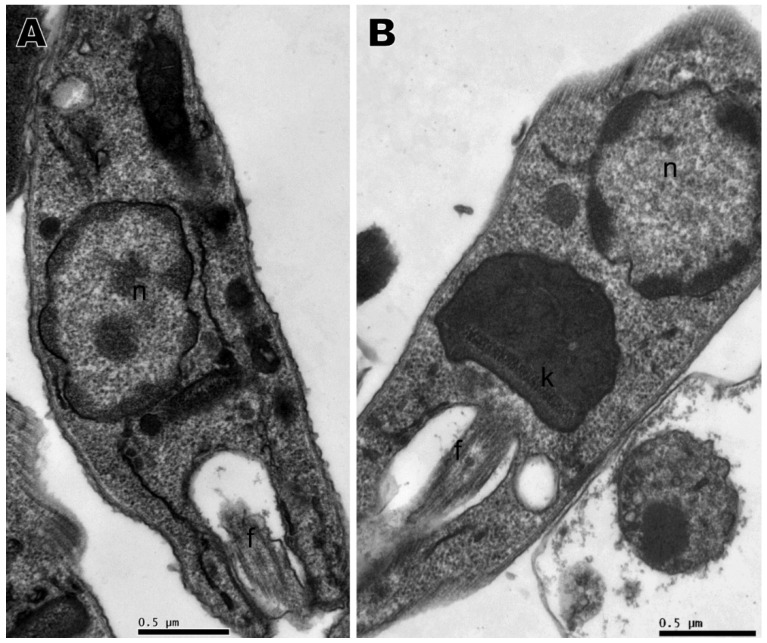
Transmission electron microscopy of *Leishmania amazonensis* promastigotes. (**A**) Untreated parasites. (**B**) Parasites treated for 24 h with 1,4-disubstituted-1,2,3-triazole **4** at 15.68 µM presenting kinetoplast swelling and a change in chromatin distribution. n: nucleus, f: flagellum, k: kinetoplast.

**Figure 5:**
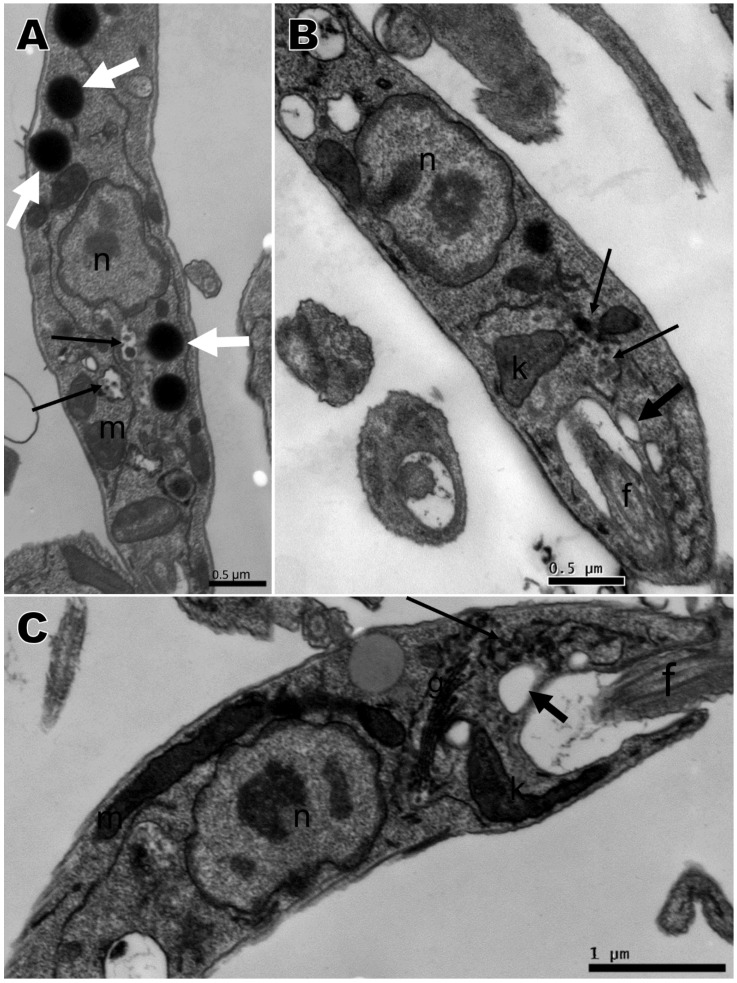
Ultrastructural alterations of *Leishmania amazonensis* promastigote forms treated for 24 h with 1,4-disubstituted-1,2,3-triazole **2** at 8.85 μM. (**A**–**C**) Lipid corpuscles (white arrows), an electron-dense vesicles free or within vacuoles in the cytoplasm (thin arrows), and small vacuoles near the flagellar pocket (black arrows). n: nucleus, m: mitochondria, k: kinetoplast, f: flagellum.

**Figure 6:**
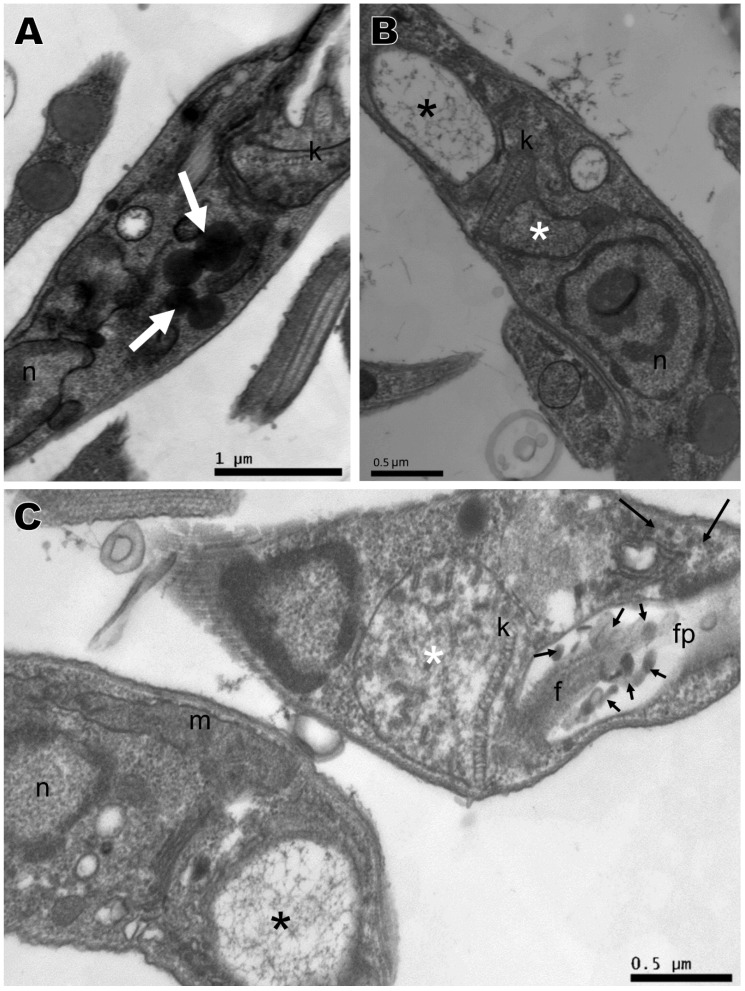
Ultrastructural alterations of *Leishmania amazonensis* promastigote forms treated for 24 h with 1,4-disubstituted-1,2,3-triazole **5** at 8.81 μM. (**A**–**C**) Lipid corpuscles (white arrows), filamentous electron-dense material within the vacuole (black asterisks), kinetoplast swelling with breakdown of mitochondrial cristae (white asterisks), and electron-dense material within the flagellar pocket (short thin arrows). k: kinetoplast, n: nucleus, f: flagellum, fp: flagellar pocket, m: mitochondria.

**Figure 7:**
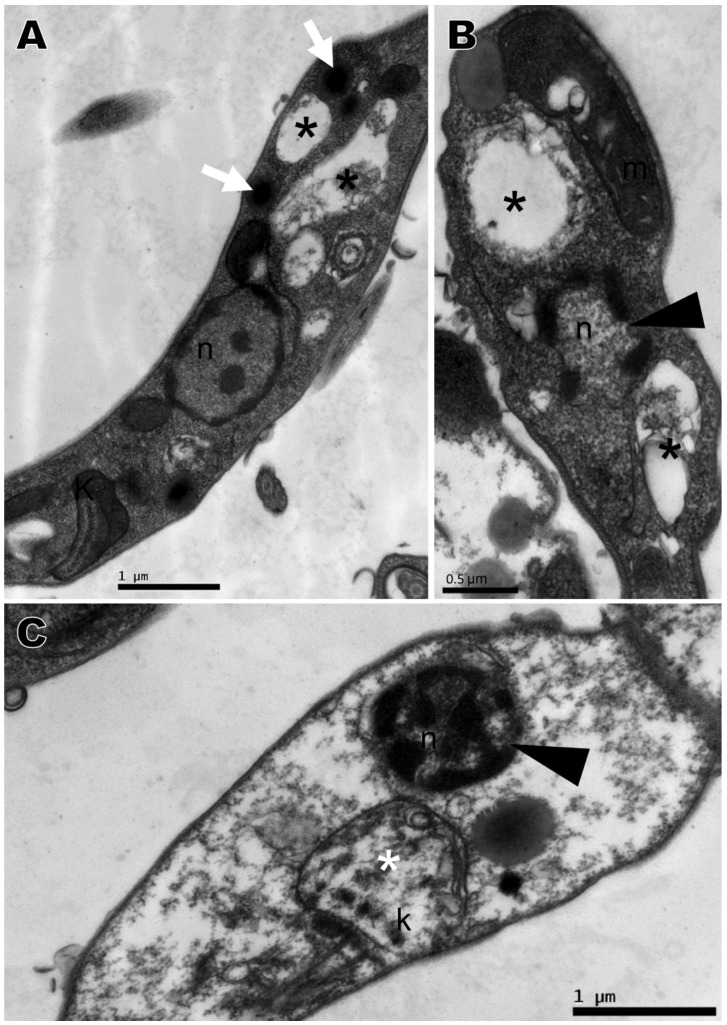
Ultrastructural alterations of *Leishmania amazonensis* promastigote forms treated for 24 h with 1,4-disubstituted-1,2,3-triazole **6** at 14.64 μM. (**A**–**C**) Lipid corpuscles (white arrows), large vacuoles containing electron-dense material (black asterisks), nucleus with discontinued nuclear membrane and change in the nuclear chromatin (arrowhead), and trace of kinetoplast with electron-density loss (white asterisks), loss in cytoplasm organelles. n: nucleus, k: kinetoplast, m: mitochondria.

Transmission electron microscopy was performed with the compounds that presented the best activity against promastigotes, namely compounds **2**, **4**, **5**, and **6**, based on IC_50_. No changes were observed in the nucleus, mitochondria, flagellum, kinetoplast, or any other organelle in parasites without treatment, which displayed normal morphology (Figure 4A). All four compounds induced ultrastructural alterations in the promastigote forms of *L. amazonensis* after 24 h of treatment. Parasites treated with compound **4** presented swelling of the kinetoplast and a change in chromatin distribution (Figure 4B). Compound **2** induced an increase in lipid corpuscles (white arrows), electron dense vesicles inside small vacuoles in the cytoplasm (thin arrow) (Figure 5A) or free vesicles with electron-dense material in the cytoplasm (thin arrows) and small vacuoles near the flagellar pocket (black arrows) (Figure 5B,C). Parasites treated with compound **5** exhibited lipid corpuscles (white arrows) (Figure 6A), extension of the kinetoplast containing granular material (white asterisk) (Figure 6B) or a swollen kinetoplast (white asterisk) as well as swollen mitochondria with deranged cristae (Figure 6C), vacuoles containing filamentous material (black asterisks) (Figure 6B,C), a flagellar pocket with electron dense material in a circular or rod shape (short thin arrow), and vesicles near the flagellar pocket (thin arrow) (Figure 6C), undefined nuclear membrane (Figure 6B,C) and nuclei with altered chromatin distribution (Figure 6A–C). Compound **6,** as well as inducing lipid corpuscles (white arrows) (Figure 7A), large vacuoles containing electron-dense material (black asterisks) (Figure 7A,B), and swollen mitochondria with degenerated regions (Figure 7B), also exhibited a parasite in the degeneration stage, a trace of kinetoplast with electron-density loss (white asterisks) and nuclear alterations, such as the discontinuity of the nuclear membrane and pyknotic chromatin (arrowhead), and the loss of cytoplasm organelles, highlighting its antileishmanial potential (Figure 7C).

### 2.4. Nitrite Quantification in Supernatant of Peritoneal Macrophages Treated with 1,4-Disubstituted-1,2,3-Triazole Compounds

**Figure 8:**
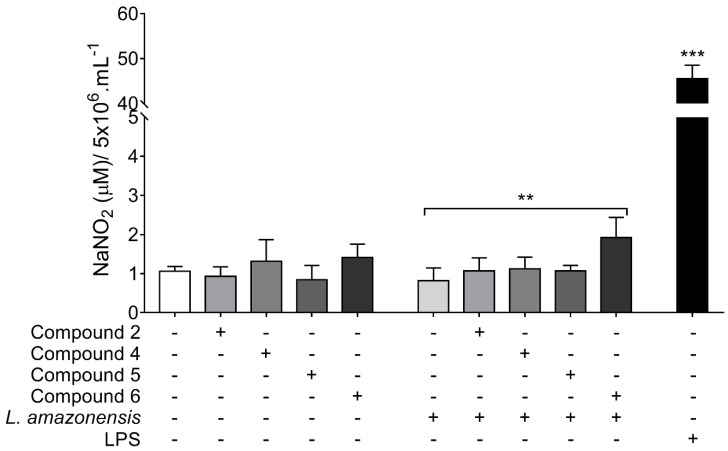
Nitrite quantification in supernatant of BALB/c peritoneal macrophages treated with 1,4-disubstituted-1,2,3-triazole compounds. Cells are stimulated (or not stimulated) with *Leishmania amazonensis* and treated for 48 h with compound **2**, **4**, or **5** at 37.5 μM, or with compound **6** at 18.7 μM. Data represents mean ± SD of at least three independent experiments realized at least in quadruplicate. ** *p* = 0.0039, *** *p* = 0.0001 when compared to untreated and non-infected cells, or between brackets by Kruskal-Wallis followed by Dunn’s multiple comparisons test.

As shown in Figure 8, only BALB/c peritoneal macrophages stimulated with *L. amazonensis* and treated with compound **6** exhibited high nitrite levels when compared to untreated and stimulated macrophages. The nitrite level was 2.3 fold higher in macrophages stimulated and treated with compound **6** than in untreated infected macrophages.

### 2.5. In Silico Drug-Likeness Prediction

In silico drug-likeness predictions were performed with compounds **2**, **4**, **5**, and **6,** which presented the best antileishmanial activity. Table 2 depicts the physicochemical, drug-likeness, and medicinal chemistry properties for 1,4-disubstituted-1,2,3-triazole compounds and miltefosine, the only available drug against leishmaniasis that is administered orally. The physicochemical characteristics of the topological polar surface area (TPSA) and lipophilicity of compound **6** were similar to miltefosine. The other compounds exhibited high polarity, except for compound **4**, and lipophilicity inferior to compound **6**. The drug-likeliness rules, such as the Lipinski rule of five and the Veber Ghose, Muegge, and Egan rules, were applied to the compounds. From the data, almost all the compounds obeyed the rules, with the exception of compound **2** in the Ghose rule, due to its high molecular weight. In medicinal chemistry property prediction, two complementary pattern recognition methods were used which allow the identification of potentially problematic fragments: pan-assay interference compounds (PAINS); and Brenk filters. None of the compounds created an alert by the PAINS evaluation, but all presented at least one problematic moiety by Brenk analysis. The lead-likeness criteria, which predicts if a molecular entity is suitable for optimization, identified only compound **2** as a good lead compound. On the other hand, the low value of synthetic accessibility indicates that all four compounds could be easily synthesized. These results indicate that 1,4-disubstituted-1,2,3-triazole compounds have drug-like properties.

### 2.6. In Silico Pharmacokinetics and Toxicity Prediction

Pharmacokinetic properties and toxicity parameters were predicted for compounds **2**, **4**, **5**, and **6** and for miltefosine (Table 3). All the 1,4-disubstituted-1,2,3-triazole compounds displayed moderate water solubility, high intestinal absorption, P-glycoprotein I and II inhibition and low skin permeability, while compounds **4** and **6** were predicted as P-glycoprotein substrates, and only compound **4** showed high Caco-2 permeability. Human steady state volume of distribution (ssVD) was moderate for compound **2** and low for the other compounds, and only compound **6** was predicted to be readily distributed to the brain through the blood-brain barrier (BBB) and penetrate the central nervous system (CNS). All 1,4-disubstituted-1,2,3-triazole compounds revealed metabolization by isoform CYP3A4, but not by CYP2D6, and all the compounds exhibited the predicted inhibition of at least three isoforms of cytochrome P450, with compound **6** inhibiting four isoforms. Compound **6** presented the lowest total clearance of all, and no 1,4-disubstituted-1,2,3-triazole compound was predicted as a renal organic cation transporter 2 (OCT2) substrate. In toxicity parameters prediction, all 1,4-disubstituted-1,2,3-triazole compounds showed hepatotoxicity, high human maximum tolerated dose (>0.477 log mg/kg/day), toxicity to flathead minnows and *Tetrahymena piriformis*, and human ether-a-go-go (hERG) II inhibition, but not hERG I inhibition or skin sensitization. Compounds **5** and **6** presented mutagenic potential and potential carcinogenic action by *Salmonella*/microsome mutagenicity assay (AMES) prediction. While compound **4** exhibited the lowest oral rat acute toxicity, it also exhibited the highest value relative to the lowest dose of a compound that resulted in an observed adverse effect (LOAEL) in oral rat chronic toxicity, followed by compound **6**.

## 3. Discussion

The present study demonstrated the in vitro antileishmanial activity of 1,4-disubstituted-1,2,3-triazole compounds, its effects on the ultrastructure of the promastigote form of *L. amazonensis*, and described the drug-likeness, pharmacokinetic and toxicity properties of the compounds by in silico prediction.

Several authors have reported the use of different 1,2,3-triazole derivatives as potential antileishmanial agents [27,28,29]. According to our results on activity against promastigote forms, compounds **2**, **4**, **5**, and **6** showed significant results. It was noted that in some of these compounds there are important pharmacophoric groups in addition to the triazole moiety, such as sulfonylhydrazone and hydrazone, also known for their antileishmanial activity [30,31]. Compound **2**, which has two triazole moieties, exhibited activity against promastigotes and intracellular amastigotes of *L. amazonensis*.

Evaluation of the intracellular amastigote revealed that compound **6** had the best activity of the compounds analyzed. In addition to triazole, compound **6** contains another important pharmacophoric group, the arylhydrazone. Hydrazones are important groups, known for their leishmanicidal properties and presence in many compounds used as antileishmanial agents [31,32]. The arylhydrazone may be related to the greater activity exhibited by compound **6** in comparison to the other compounds analyzed.

The highest cytotoxicity against BALB/c peritoneal macrophages was exhibited by compounds **2**, **4**, and **5**. This may be related to the presence of the aldehyde group in compounds **2** and **4**, and the sulfonyl group in compound **5**. It is known that sufficiently electrophilic groups, such as the aldehyde and sulfonyl groups, can react with DNA [33], which may explain the toxicity displayed by these compounds. In addition, the sulphonyl hydrazine derivatives are associated with the inhibition of mammal cells enzymes [25,34], such as carbonic anhydrase, an enzyme that plays an important role in pH regulation [25], which may be related to the higher cytotoxicity of compound **5** and its sulfanyl moiety. Compound **6,** meanwhile, had the lowest cytotoxicity, possibly associated with the presence of the arylhydrazone group. Several studies have described the use of hydrazone derivatives with leishmanicidal activity, reporting their good selectivity rates and low cytotoxicity [31,32,35].

The cytotoxicity result, associated with activity against intracellular amastigotes, revealed compound **6** to have the highest selectivity for parasites among the compounds, with SI even better than miltefosine. The selectivity index is an established criterion for the identification of potential compounds against infectious diseases. The Japanese Global Health Innovative Technology and the Drugs for Neglected Diseases initiative agreed that the SI of a lead compound should be greater than the 10-fold selectivity window for cytotoxicity using a mammalian cell [36]. Compound **6** falls within this criteria, and has thus been classified as a hit compound.

The transmission electron microscopy of *L. amazonensis* promastigotes treated with 1,4-disubstituted-1,2,3-triazole compounds revealed various alterations in the parasite ultrastructure. One remarkable change observed was the alterations in mitochondria, such as the swelling of the kinetoplast and the breakdown of mitochondrial cristae. These mitochondrial alterations were also observed in *L. amazonensis* promastigotes treated with a triazole hybrid of neolignanes [37] and with ravuconazole [38], a triazole antifungal drug. These ultrastructural alterations are related to dysfunctional mitochondria. It was reported that 1,2,3-triazole derivatives induce mitochondrial alteration through an increment in ROS and depolarization of the mitochondrial membrane potential of *L. amazonensis* promastigotes [22].

Another ultrastructural alteration described in literature that we observed in parasites treated with 1,4-disubstituted-1,2,3-triazole compounds was the presence of lipid corpuscles. These lipid inclusions were associated with vesicles with electron-dense material in the cytoplasm and near the flagellar pocket, as well as with the presence of material inside the flagellar pocket. All these alterations may be related to the alteration in lipid biosynthesis, which induces the accumulation of lipid bodies [37,38] and promotes the formation of vesicles in the cytoplasm, as a consequence of drug action or the indication of the exacerbated production of proteins by cells in an attempt to survive, resulting in increased activity in the region of the exocytic flagellar pocket as a result of the abnormal secretion of lipids [39]. These alterations have also been described in other study using *L. amazonensis* promastigotes treated with ergosterol synthesis inhibitors [40]. *Leishmania* parasites produce ergosterol-related sterols by a biosynthetic pathway similar to that which operates in the pathogenic fungi, and their growth is susceptible to sterol biosynthesis inhibitors [41], a plausible mechanism of action of the triazole compounds.

Nuclear alterations, such as chromatin condensation and the discontinuity of the nuclear membrane, have already been observed and described in *L. amazonensis* promastigotes treated with triazole compounds [37], and were observed in parasites treated with compound **6**, showing that this compound has potent antileishmanial activity in vitro.

Nitrite quantification was used to indirectly quantify nitric oxide (NO) production in BALB/c peritoneal macrophages. Although all the 1,4-disubstituted-1,2,3-triazole compounds increased nitrite levels in the peritoneal macrophage, compound **6** was able to induce NO production in macrophages infected with *L. amazonensis*. NO is a short-lived and freely diffusible gas that originates from the conversion of l-arginine to l-citrulline, by NO synthase (NOS) enzyme [42]. In infected host organisms, NO demonstrates antimicrobial and immunostimulatory (proinflammatory) effects, contributing to the killing of intracellular parasites like *Leishmania* [43,44]. The increase in NO production by compound **6** evidences the probability of macrophage immunomodulation as one of its effector mechanisms against intracellular amastigotes.

Further to in vitro studies, we also carried out in silico analyses. Studies using in silico methodologies are tools that can be used in drug development, giving physical-chemistry, drug-likeness, pharmacokinetic and toxicity parameters, which may be helpful for further studies. Medicinal chemistry, for example, analyzed the physicochemical structure of compounds and predicted the presence or absence of a problematic moiety, lead-likeness, and synthetic accessibility in 1,2,3-triazole [45].

Lipinski’s rule of five is a drug-likeness parameter widely used in in silico analysis for the evaluation of physio-chemical properties that would make a compound behave as an orally active drug in humans. This rule states that molecular mass should be less than 500 Daltons, have no more than five H-bond donors and no more than 10 H-bond acceptors, and a calculated Log P (CLogP) no greater than five (or MLogP > 4.15). Compounds that violate more than two of Lipinski’s rules may encounter problems in the first step of absorption, such as poor solubility and intestinal permeability, interfering in oral bioavailability [46]. For an even more rigorous evaluation, four drug-likeness in silico predictions were performed—the Ghose (Amgen), Veber (GSK), Egan (Pharmacia), and Muegge (Bayer) methods [45].

Several specialized prediction models were also compiled with the pharmacokinetic parameters, evaluating the individual absorption, distribution, metabolization, and excretion behaviors of the compounds under investigation. The same was performed with the toxicity parameters. The pkCSM [47] and SwissADME [45] tools were used throughout the in silico study we used, comparing and complementing the results to achieve a robust outcome, targeting the desired characteristics of a new drug for leishmaniasis. Due to the low skin permeability, the 1,4-disubstituted-1,2,3-triazole compounds, in the same way as miltefosine, are not strong candidates for a topical formulation aimed at cutaneous leishmaniasis treatment. However, considering all the in silico prediction analysis, the compound **(6)**, that presented promising antileishmanial activity in vitro, exhibited similar or even better results than miltefosine and is a good candidate for further in vivo studies against *Leishmania* parasites.

## 4. Materials and Methods

### 4.1. Reagents

Dimethyl sulfoxide (DMSO), Schneider’s Insect medium, streptomycin, 3-(4,5-dimethylthiazol-2-yl)-2,5-diphenyltetrazolium bromide (MTT), miltefosine, Brewer thioglycollate medium, RPMI 1640, glutaraldehyde, sodium-cacodylate, EPON 812, sulfanilamide, N-(1-naphthyl) ethylenediamine, H_3_PO_4_, osmium tetroxide, potassium ferrocyanide, calcium chloride, uranyl acetate, lead citrate, sodium citrate and acetone were purchased from Sigma, St Louis, MO, USA. Fetal bovine sera and penicillin were purchased from Gibco, Gaithersburg, MD, USA. Giemsa’s azur-eosin-methylene blue was purchased from MERK, Darmstadt, Germany.

### 4.2. Triazole Compounds

The 1,4-disubstituted-1,2,3-triazole derivatives **1**–**10** were previously synthesized by copper-catalyzed azide-alkyne cycloaddition reaction, as described by Silva et al. 2019 [24]. All the compounds were structurally characterized by the ^1^H NMR, ^13^C NMR and mass spectrometry techniques. Stock solutions were prepared in DMSO with a final concentration that never exceeded 1% DMSO, which is not toxic to either the parasite and mammalian cells.

### 4.3. Parasites

*Leishmania amazonensis* (MHOM/BR/76/MA-76) promastigote forms of were cultured at 26 °C in Schneider’s Insect medium added with 10% fetal bovine sera (FBS), 100 U/mL of penicillin and 100 µg/mL of streptomycin. Parasite cultures with a maximum of seven in vitro passages were used.

### 4.4. Activity against Promastigote Forms

*L. amazonensis* promastigote forms obtained from a 2–4-day-old culture were placed in 96-well plates with varied concentrations of triazole compounds obtained by serial dilutions 1:2 (300 to 1.17 µM), at a final volume of 100 µL per well, for 72 h. Blanks composed by wells without parasites and wells with parasites plus DMSO 1% only were used as controls. A modified colorimetric method with tetrazolium-dye MTT was used to evaluate parasite viability [48,49]. A quantity of 10 µL of MTT (5 mg/mL) was added to each well. After five hours, 150 µL of DMSO was added to each well to dissolve the formazan crystals. Absorbance was read on a spectrophotometer at a wavelength of 570 nm. The data was normalized and the results were used to calculate the IC_50_ (50% inhibition of parasite growth). Miltefosine was used as a reference drug.

### 4.5. Animals

The local Ethics Committee on Animal Care and Utilization authorized all the procedures with animals (CEUA/IOC – L053/2016, December 28, 2016). BALB/c female mice 4–6 weeks old purchased from the Instituto de Ciência e Tecnologia em Biomodelos, Instituto Oswaldo Cruz, Rio de Janeiro were used in the study. The animals were maintained under pathogen-free conditions and handled in accordance with the National Council for Control of Animal Experimentation (Conselho Nacional de Controle de Experimentação Animal; CONCEA).

### 4.6. Cell Culture

The peritoneal macrophages were obtained from BALB/c mice elicited with 3 mL Brewer thioglycollate medium for 72 h, intraperitoneal. After euthanasia, cells were obtained by peritoneal washing with PBS, centrifuged at 4000 rpm for 5 min, and suspended in RPMI 1640 medium plus 10% FBS, 100 U/mL of penicillin and 100 µg/mL of streptomycin [50]. The cells were immediately used in experiments and maintained at 37 °C and 5% CO_2_.

### 4.7. Cytotoxicity Assay

BALB/c peritoneal macrophages were cultured in 96-well plates (5 × 10^5^ cells/mL) with different concentrations of triazole compounds obtained by serial dilutions 1:2 (600 to 2.34 µM), or miltefosine (50 to 0.19 µM) up to a final volume of 100 µL per well, at 37 °C and 5% CO_2_. Blanks were composed by wells without cells and wells with cells plus 1% of DMSO only. Cell viability was carried out by the modified MTT colorimetric method as described before [51]. Tem microliters of MTT at 5 mg/mL was added to each well and incubated for two hours at 37 °C and 5% CO_2_. The plate was then centrifuged for five minutes at 1500 rpm, the supernatants were removed and the formazan crystals solubilized with 100 μL of DMSO in a shaker-plate for 10 min. The absorbance was determined in a spectrophotometer at a wavelength of 570 nm. Cytotoxicity was demonstrated as a percentage, and the concentration inhibiting 50% of cell growth (CC_50_) was calculated using the GraphPad Prism 7.00 (GraphPad Software, San Diego, CA, USA) software package.

### 4.8. Activity against Intracellular Amastigote and Selectivity Index

Activity against the intracellular amastigotes was carried out in 24-well plates with coverslips with peritoneal macrophages (5 × 10^5^ cells/well). Cells were infected with *L. amazonensis* promastigote forms using a ratio of 10:1 parasite/cell. After 6 h after infection the cells were washed three times with PBS to remove non-internalized parasites. Infected cells were treated with different concentrations of triazole compounds obtained by serial dilutions 1:2 (37.5–2.3 μM) or miltefosine (25–1.56 μM) for 24 h. The infected and treated cells adhered to the coverslips were fixed with Bouin, stained with Giemsa’s azur-eosin-methylene blue and examined by light microscopy. The intracellular number of amastigotes of 200 cells were normalized and used to calculate the IC_50_. The percentage of infected cells was determined by number of infected cells divided by two. The mean number of amastigotes per cell was determined using the number of intracellular amastigotes counted in 200 cells divided by the number of infected cells. The selectivity index was calculated from the ratio of CC_50_ versus the IC_50_ for intracellular amastigotes [52].

### 4.9. Transmission Electron Microscopy

The promastigote forms of *L. amazonensis* were treated for 24 h with IC_50_ of the 1,2,3-triazole compounds which exhibited the best activity against the promastigotes. The parasites were fixed with 2.5% glutaraldehyde in a 0.1 M sodium-cacodylate buffer, pH 7.2 overnight. Then, parasites were washed three times with 0.1 M sodium-cacodylate buffer, post-fixed in a solution composed by 1% osmium tetroxide, 0.8% potassium ferrocyanide, and 5 mM calcium chloride, dehydrated in graded acetone and embedded in EPON 812. Ultrathin sections were obtained from 100 nm cuts in Sorvall MT 2-B (Porter Blum) ultramicrotone (Sorvall, Newtown, CT, USA) stained with 5% uranyl acetate aqueous solution and lead citrate (1.33% lead nitrate and 1.76% sodium citrate), and examined in a transmission electron microscope JEM-1011 (JEOL, Tokyo, Japan) operating at 80 kV [53].

### 4.10. Nitrite Quantification in Macrophages Stimulated with L. amazonensis and Treated with Triazoles

BALB/c peritoneal macrophages at 5 × 10^6^ cells/mL were stimulated with *L. amazonensis* promastigotes (10:1 parasites:cell) for one hour, and treated with different concentrations of triazole compounds for 48 h. Nitrite quantification of the supernatant of the cells was performed with Griess reagent [54]. Then, 50 μL of culture supernatant were added to 50 μL of Griess reagent (25 μL of sulfanilamide 1% in 2.5% H_3_PO_4_ solution and 25 μL of N-(1-naphthyl) ethylenediamine 0.1% solution) in 96-well plates and read after 10 min at 570 nm on the spectrophotometer. The nitrite concentrations were obtained from the standard curve of sodium nitrite (100 to 0.3 μM) [43].

### 4.11. In Silico Pharmacokinetics Prediction

The structures of 1,2,3-triazole compounds were drawn using ChemDraw software (version Ultra 12.0, PerkinElmer Informatics, Waltham, MA, USA) and were converted into a single database file SMILES. In silico physicochemical, drug-likeness, pharmacokinetics and toxicity properties were assessed with the pkCSM [47] and SwissADME [45] web tools.

### 4.12. Statistical Analyses

A nonlinear regression fit curve of concentration log versus normalized response originated the IC_50_ and CC_50_. The data were expressed as mean ± S.D. GraphPad Prism 7.00 were used to perform the analyses and differences were considered significant when *p* < 0.05.

## 5. Conclusions

The 1,4-disubstituted-1,2,3-triazole compounds were evaluated against *L. amazonensis*, with compound **6** exhibiting the best activity against the promastigote and intracellular amastigote forms, altering all parameters of in vitro infection. This compound also exhibited low cytotoxicity to macrophages and a high selectivity index to parasites over cells. Ultrastructural analysis showed that 1,4-disubstituted-1,2,3-triazole treatment induces mitochondrial alterations, such as swelling of the kinetoplast and the breakdown of the mitochondrial cristae, suggesting its dysfunction, and lipid bodies inclusion with an increase in exocytic activity that may be related to lipid biosynthesis inhibition. Compound **6** was able to enhance 2.3-fold the nitrite levels in the stimulated macrophage. In silico pharmacokinetics prediction analysis of compound **6** revealed that it is not recommended for topical formulation aimed at cutaneous leishmaniasis treatment. The other properties, however, exhibited results that were similar or even better than miltefosine, making it a promising candidate for further in vivo studies against *Leishmania* parasites.

## Figures and Tables

**Table 1:** Activity against promastigotes and intracellular amastigotes of *Leishmania amazonensis*, cytotoxicity in BALB/c peritoneal macrophages, and selectivity index after 24 h of treatment with 1,4-disubstituted-1,2,3-triazole compounds.

Compounds	IC_50_ (µM)		CC_50_ (µM)	SI
	Promastigote	Intracellular Amastigote		
**1**	53.70 ± 8.467	–	221.20 ± 4.824	–
**2**	**8.85 ± 3.436**	37.92 ± 3.350	248.46 ± 2.519	6.55
**3**	50.93 ± 4.571	–	307.73 ± 4.071	–
**4**	**15.68 ± 5.418**	149.8 ± 6.367	144.80 ± 4.100	0.96
**5**	**8.81 ± 3.933**	32.31 ± 2.256	58.01 ± 2.647	1.79
**6**	**14.64 ± 4.392**	**17.78 ± 3.257**	547.88 ± 3.256	**30.81**
**7**	62.98 ± 5.878	–	412.00 ± 4.917	–
**8**	>300	–	>600	–
**9**	>300	–	>600	–
**10**	>300	–	>600	–
Miltefosine	8.56 ± 0.695	11.615 ± 1.790	152.61 ± 3.855	13.13

IC_50_: inhibitory concentration of 50% parasites. CC_50_: cytotoxicity concentration of 50% cells. SI: selectivity index calculated from the ratio of CC_50_ versus the IC_50_ for intracellular amastigotes. –: not determined. Data represent mean ± standard deviation of three experiments performed at least in triplicate.

**Table 2:** Predicted physicochemical, drug-likeness, and medicinal chemistry properties for 1,4-disubstituted-1,2,3-triazole compounds and miltefosine.

Property/Model Name	Compounds				
	2	4	5	6	Miltefosine
**Physicochemical**					
Molecular Weight	480.484	333.347	447.52	369.429	407.576
# Rotatable bonds	10	7	8	7	20
# H-bond acceptors	8	5	6	4	4
# H-bond donors	0	0	1	1	0
Surface Area	205.260	144.553	185.782	162.657	168.579
TPSA (Å^2^)	114.02	66.24	106.85	64.33	68.40
Lipophilicity (Log P_o/w_)	2.87	2.63	3.24	3.70	3.83
**Drug-likeness**					
Lipinski	Yes; 0 violation	Yes; 0 violation	Yes; 0 violation	Yes; 0 violation	Yes; 0 violation
Ghose	No; 1 violation: MW > 480	Yes	Yes	Yes	No; 2 violations: WLOGP > 5.6, #atoms > 70
Veber	Yes	Yes	Yes	Yes	No; 1 violation: Rotors > 10
Egan	Yes	Yes	Yes	Yes	No; 1 violation: WLOGP > 5.88
Muegge	Yes	Yes	Yes	Yes	No; 2 violations: XLOGP3 > 5, Rotors > 15
**Medicinal chemistry**					
PAINS	0 alert	0 alert	0 alert	0 alert	0 alert
Brenk	1 alert: aldehyde	2 alerts: aldehyde, triple bond	1 alert: imine	1 alert: imine	2 alerts: phosphor, quaternary nitrogen
Lead-likeness	No; 2 violations: MW > 350, Rotors > 7	Yes	No; 3 violations: MW > 350, Rotors > 7, XLOGP3 > 3.5	No; 2 violations: MW > 350, XLOGP3 > 3.5	No; 3 violations: MW > 350, Rotors > 7, XLOGP3 > 3.5
Synthetic accessibility	3.44	3.00	3.52	3.25	4.67

#: number, TPSA: topological polar surface area, PAINS: pan-assay interference compounds, MW: molecular weight.

**Table 3:** In silico pharmacokinetics and toxicity properties of 1,4-disubstituted-1,2,3-triazole compounds and miltefosine.

Property	Model Name	Compounds				
		2	4	5	6	Miltefosine
Absorption	Water solubility (log mol/L)	−4.143	−4.327	−5.487	−5.382	−6.149
	Caco-2 permeability (log Papp in 10^−6^ cm/s)	0.538	1.178	0.776	0.516	1.049
	Intestinal absorption – human (% Absorbed)	95.298	100	93.529	92.221	92.021
	Skin Permeability (log Kp)	−2.735	−2.641	−2.739	−2.732	−2.721
	P-glycoprotein substrate	No	No	Yes	Yes	No
	P-glycoprotein I inhibitor	Yes	Yes	Yes	Yes	Yes
	P-glycoprotein II inhibitor	Yes	Yes	Yes	Yes	Yes
Distribution	Human ssVD (log L/kg)	0.068	−0.284	−0.241	−0.236	0.355
	BBB permeability (log BB)	−1.675	0.039	−0.843	0.393	−0.176
	CNS permeability (log PS)	−3.531	−2.579	−2.455	−1.997	−3.191
Metabolism	CYP2D6 substrate	No	No	No	No	No
	CYP3A4 substrate	Yes	Yes	Yes	Yes	Yes
	CYP1A2 inhibitor	No	Yes	No	Yes	No
	CYP2C19 inhibitor	No	Yes	Yes	Yes	No
	CYP2C9 inhibitor	Yes	Yes	Yes	Yes	No
	CYP2D6 inhibitor	No	No	No	No	No
	CYP3A4 inhibitor	Yes	No	Yes	Yes	No
Excretion	Total Clearance (log mL/min/kg)	0.386	0.35	0.671	0.199	1.112
	Renal OCT2 substrate	No	Yes	No	No	No
Toxicity	AMES toxicity	No	No	Yes	Yes	No
	Human max. tolerated dose (log mg/kg/day)	0.796	0.692	0.844	0.898	0.211
	hERG I inhibitor	No	No	No	No	No
	hERG II inhibitor	Yes	Yes	Yes	Yes	Yes
	Oral Rat Acute Toxicity LD50 (mol/kg)	3.024	1.949	2.747	2.599	2.655
	Oral Rat Chronic Toxicity LOAEL (log mg/kg bw/day)	0.653	1.39	0.462	0.99	0.233
	Hepatotoxicity	Yes	Yes	Yes	Yes	Yes
	Skin Sensitization	No	No	No	No	Yes
	*T pyriformis* toxicity (log µg/L)	0.285	0.474	0.294	0.327	0.311
	Minnow toxicity (log mM)	−1.708	−1.322	−2.1	−3.393	−1.839

VDss: steady state volume of distribution, BBB: brain-blood barrier, CNS: central nervous system, OCT2: organic cation transporter 2, AMES: *Salmonella*/microsome mutagenicity assay, hERG: human ether-a-go-go gene, LOAEL: lowest dose of a compound that resulted in an observed adverse effect.

## Abbreviations

WHO	World Health Organization
IC_50_	inhibitory concentration of 50%
CC_50_	cytotoxicity concentration of 50%
SI	selectivity index
TPSA	topological polar surface area
PAINS	pan-assay interference compounds
VDss	steady state volume of distribution
CNS	central nervous system
BBB	blood-brain barrier
hERG	human ether-a-go-go gene
AMES	*Salmonella*/microsome mutagenicity assay
LOAEL	lowest dose of a compound that results in an observed adverse effect
OCT2	organic cation transporter 2,
DMSO	dimethyl sulfoxide
MTT	3-(4,5-dimethylthiazol-2-yl)-2,5-diphenyltetrazolium bromide
